# Predicting extrauterine growth restriction at the parenteral-enteral nutrition transition: a model for risk identification in very preterm infants

**DOI:** 10.3389/fnut.2026.1788647

**Published:** 2026-09-03

**Authors:** Zhenyuan Wu, Dan Zhou, Yanhua Hu, Rong Chen, Min Huang, Baoquan Zhang, Wenlong Xiu

**Affiliations:** 1 Department of Neonatology, Fujian Maternity and Child Health Hospital College of Clinical Medicine for Obstetrics & Gynecology and Pediatrics, Fujian Medical University, Fuzhou, China; 2 Fujian Clinical Research Center for Maternal-Fetal Medicine, Fuzhou, Fujian, China; 3 National Key Obstetric Clinical Specialty Construction Institution of China, Fuzhou, Fujian, China

**Keywords:** Fenton 2013, logistic regression, machine learning, restricted growth, very preterm

## Abstract

**Introduction:**

This study aims to identify the key predictors of longitudinal extrauterine growth restriction (EUGR) in very preterm infants during the parenteral-to-enteral nutrition transition and subsequently develop an effective predictive model for early clinical identification.

**Methods:**

A total of 260 infants born at 28–31^+6^ weeks of gestation were included in this retrospective study. Perinatal and early postnatal clinical data were collected. The univariate regression analysis (*p* < 0.1) and LASSO algorithms were employed to comprehensively screen for variables. The screened variables were utilized to construct the optimal model. The development of an optimal predictive model involved performance comparison and validation across five algorithms, including logistic regression and four machine learning algorithms. This process was coupled with an explanation of machine learning algorithms using the Shapley Additive exPlanations (SHAP) to ensure interpretability.

**Results:**

The study identified six key predictors: gestational age, sex, time to regain birth weight, gestational diabetes, hypertensive disorders of pregnancy and blood urea nitrogen. Among the models compared, the logistic regression model exhibited the best performance, and the nomogram developed from this model demonstrated strong predictive efficacy.

**Conclusion:**

In this study, we developed and internally validated a prediction model for longitudinal EUGR in very preterm infants specifically during the parenteral-to-enteral transition. The model demonstrated robust predictive performance, strong clinical utility, and exceptional interpretability. It offers a practical tool for risk stratification during the parenteral-to-enteral transition, enabling clinicians to identify high-risk infants who may benefit from timely, personalized nutritional support.

## 1 Introduction

Very preterm infants, defined by the World Health Organization (WHO) as those born at 28 to <32 weeks of gestation (1, 2), present a significant clinical challenge due to profound organ immaturity, which underlies their substantially elevated risks of mortality and major morbidities (3). In China, this subgroup is of particular importance: data from the Chinese Neonatal Network (CHNN) show that 88.3% of infants born at <32 weeks were delivered between 28 and 31^+6^ weeks’ gestation, constituting the vast majority of very preterm births and representing a key population for neonatal intensive care (4).

The 28–31^+6^ week gestational age stratum accounts for a substantial proportion of preterm births, making targeted nutritional interventions in this group particularly impactful from both a population health and individual prognosis perspective. Moreover, this stratum possesses its own distinct nutritional and developmental characteristics, which differ from those of both extremely preterm and moderate-late preterm infants. Although advances in perinatal and neonatal care have significantly improved the survival rates of these infants, their limited intrauterine nutritional reserves and immature organ function predispose them to postnatal nutritional deficits and increased metabolic demands from comorbidities, placing them at high risk for extrauterine growth restriction(EUGR) (5, 6). EUGR has significant downstream consequences, being strongly associated with impaired childhood growth, neurodevelopmental delays, and an elevated risk of cardiovascular morbidity later in life (7–9). Consequently, the prevention of EUGR is of paramount importance for improving the overall prognosis and long-term health trajectory of these vulnerable infants.

The definition of EUGR encompasses two approaches: (1) a cross-sectional definition, defined as a weight z-score or percentile below a standard threshold at 36–40 weeks’ postmenstrual age (PMA) or at discharge; and (2) a longitudinal definition, characterized by a decline of >1 to 2 z-scores between birth and the same time points (10). The longitudinal definition of EUGR accurately captures cumulative growth trajectory abnormalities in preterm infants (11, 12). Importantly, research indicates that longitudinally defined EUGR exhibits a stronger association with poorer weight and head circumference outcomes at 24–30 months of corrected age compared to the cross-sectional definition (6, 13, 14), demonstrating its superior predictive validity for long-term growth and neurodevelopmental outcomes. Epidemiological data highlight a high burden of EUGR among very preterm infants, with cross-sectional incidence around 45% in the West and 48.4% in China. Longitudinal rates are even more pronounced, reaching approximately 59 and 74.9% in Western and Chinese cohorts, respectively (12, 15, 16). This establishes EUGR as a major clinical and public health concern for this vulnerable population globally.

Optimized nutritional management is a cornerstone of care for preterm infants, as achieving optimal early nutrition is crucial for promoting growth, shortening hospitalization, reducing morbidities, and lowering the incidence of EUGR, thereby improving survival and long-term outcome (17–20). Hence, the timely identification of infants at high risk for EUGR and the subsequent optimization of nutritional strategies are essential for effective prevention. Key modifiable strategies to operationalize this include: achieving a higher cumulative caloric intake during the first postnatal week, initiating enteral feeds early, and advancing feeding volumes more rapidly (21, 22). Predictive models, which integrate multiple risk factors to estimate disease probability enhance diagnostic accuracy, optimize prognostic assessment, and support clinical decision. This capability has led to their widespread application in neonatology, particularly for risk stratification in preterm infants concerning conditions such as bronchopulmonary dysplasia (BPD), necrotizing enterocolitis (NEC), and early-onset sepsis (EOS) (23–25). Existing predictive models for EUGR in preterm infants face several limitations that hinder their clinical utility. These include (1) a reliance of some models on cross-sectional rather than longitudinal EUGR definitions; (2) the inclusion of insufficiently comprehensive risk factors in certain models; and (3) the use of variables in some models that are unavailable in the early postnatal period, thereby limiting early risk identification and intervention. Furthermore, traditional statistical methods may be insufficient for rigorously analyzing complex, multidimensional clinical datasets. To address these gaps, this study utilized comprehensive clinical data from very preterm infants during the transition from parenteral to enteral nutrition to develop and evaluate a risk prediction model. The model development strictly adhered to the reporting guideline (26).

## 2 Materials and methods

### 2.1 Study population

This retrospective study included neonates admitted to the Department of Neonatology at Fujian Maternal and Child Health Hospital between January 2022 and December 2023. Ethical approval was obtained from the Ethics Committee of Fujian Provincial Maternity and Children’s Hospital (2024KY292). Infants were eligible for this study if they met all of the following criteria: (1) hospitalization lasting at least 2 weeks; (2) admitted to the NICU within 24 h of birth. Infants were excluded in case of (1) death, transfer, or discharge against medical advice before 36 weeks’ postmenstrual age (PMA);(2) major congenital anomalies, inherited metabolic disorders, or congenital malformations of the respiratory or gastrointestinal tract;(3) incomplete clinical records (>20% missing data), (4) discharge after a corrected gestational age >50 weeks.

### 2.2 Sample size calculation

We calculated the sample size on the basis of the events per variable (EPV) metric (27–29). In our training cohort, the incidence of EUGR was 116/182 = 0.637. Given our intention to include 4–6 predictor variables and set the EPV to 10, we calculated the required sample size via the following formula:

$\text{Sample size}=\frac{\text{Numer of Valiables}\times \mathrm{EPV}}{1-\text{Incidence Rate}}=\frac{6\times 10}{1-0.637}=65.$ In this study, the dataset was divided into train and test sets at a 7:3 ratio, resulting in a minimum required sample size of 236.

### 2.3 Data collection and variable definitions

Data were abstracted from patient charts by trained research staff adhering to standardized case report forms and entered into the dedicated study database. The following variables were collected:

Maternal data collection included: age (year), chorioamnionitis (CAM), intrauterine growth restriction (IUGR), premature rupture of membranes (PROM) > 18 h, mode of delivery, assisted reproductive technology (ART), gestational diabetes mellitus (GDM), hypertensive disorders of pregnancy (HDP).

Neonatal data collection included: (1) Basic demographic and clinical information such as gestational age(week), sex, 5-min Apgar scores, birth weight(kg), weight at days 7 and 14 (kg), weight Z-scores at birth, weight Z-scores at days 7 and 14, weight Z-scores at discharge, growth velocity(g/kg/d), length of hospitalization (d); (2) Laboratory measures were serum creatinine (SCr,μmol/L), blood urea nitrogen (BUN, mmol/L), glucose (Glu, mmol/L), and hemoglobin (HGB, g/L) on postnatal day 14; (3) Neonatal morbidities including neonatal respiratory distress syndrome (NRDS), EOS, persistent pulmonary hypertension (PPHN), patent ductus arteriosus, anemia, intraventricular hemorrhage (IVH), BPD, retinopathy of prematurity (ROP), NEC, and late-onset sepsis. (4) Treatment and nutritional support included duration of mechanical ventilation (d), time to initiation of enteral feeding (d), cumulative duration of fasting during the initial 14 days (d), time to regain birth weight (d), and intake of amino acids and lipid emulsions in total parenteral nutrition (TPN) on days 7 and 14 (g/kg), as well as milk intake on days 7 and 14 (ml).

EUGR was defined as a decrease in Z score of weight between birth and discharge of more than −1 SD using Fenton’s growth chart (6, 30). Gestational age (GA) was determined based on the last menstrual period, early pregnancy ultrasound, or calculated directly in cases of *in vitro* fertilization. Growth velocity (GV) (g/kg/d) = [1,000 × ln(Wn/W_1_)]/(Dn-D_1_), where Wn is any-time weight (g), W_1_ is birth weight (g), Dn is the age in days (d), and D_1_ is time to regain birth weight (d) (31). EOS: defined as systemic infection occurring within the first 72 h of life, including clinically suspected sepsis and culture-confirmed sepsis. IVH was defined as grades I–IV IVH detected by cranial ultrasound or magnetic resonance imaging (MRI). NEC was defined according to Bell’s criteria, stage II (32). BPD: was defined according to the 2001 NICHD criteria, as the need for supplemental oxygen for at least 28 days with a fraction of inspired oxygen (FiO₂) > 0.21 (33).

Throughout the study period, all patients received standardized care in strict accordance with the unified neonatal intensive care unit (NICU) protocols, which were rigorously implemented by the medical staff. TPN was initiated on the first day of life in the absence of contraindications, with early enteral feeding introduced as tolerated. Breast milk was preferred, supplemented by preterm formula when necessary. Enteral feeding volumes were progressively advanced while parenteral nutrition was correspondingly reduced.

### 2.4 Development and validation of the optimal model

**Figure 1:**
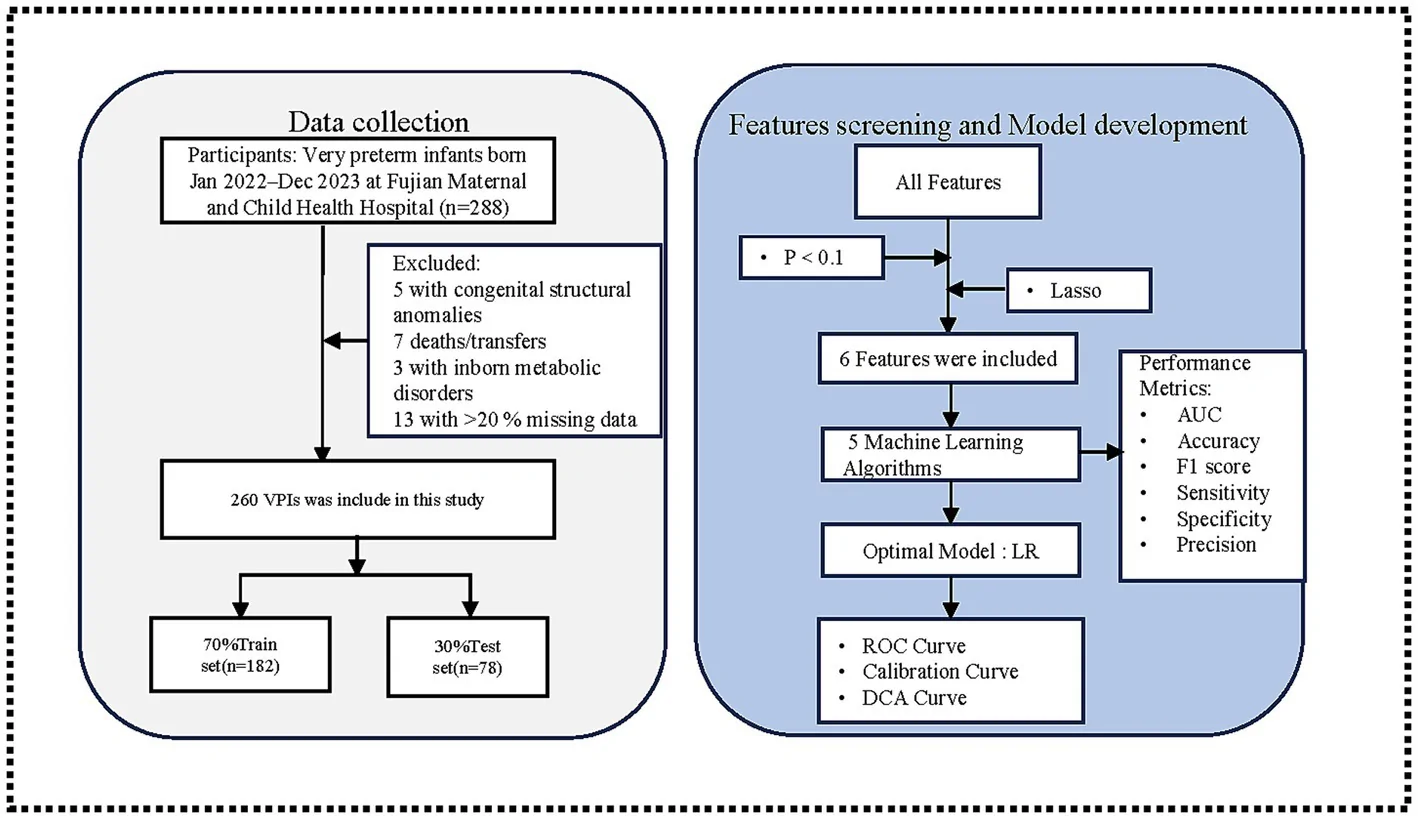
Research flow chart. LM, logistic regression model; SVM, support vector machine; KNN, *K*-nearest neighbors; LightGBM, light gradient boosting machine; LASSO, least absolute shrinkage and selection operator.

To identify potential predictors while avoiding overfitting, a rigorous two-step variable selection strategy was implemented. Initially, univariable logistic regression analyses were performed. A relatively lenient significance threshold of *p* < 0.1 was deliberately chosen to minimize the risk of excluding potentially relevant confounders or clinically significant variables that might not reach statistical significance in a univariable setting due to limited power or masking effects. Candidate variables meeting this criterion were preliminarily screened for further analysis. Subsequently, Least absolute shrinkage and selection operator (LASSO) regression with 10-fold cross-validation was applied to address multicollinearity and perform dimensionality reduction. LASSO penalizes the absolute size of regression coefficients, effectively shrinking irrelevant predictors to zero while retaining the most informative features. The optimal penalty parameter (*λ*) was determined using the minimum criteria (λ_min_), which minimizes the cross-validation error. Variables with non-zero coefficients at λ_min_ were retained for model construction. The final selected variables were used to develop machine learning models using five algorithms: light gradient boosting machine (LightGBM), categorical boosting (CatBoost), support vector machine (SVM), and k-nearest neighbors (KNN), and logistic regression model. Five-fold cross-validation was applied to ensure model stability and accuracy. Model performance was assessed by AUC, Accuracy, precision or positive prediction value, sensitivity, specificity, and F1 score. These metrics were calculated for the logistic regression model and the four machine learning models, and the model with the optimal performance was finally selected. The steps are summarized in the flow chart (shown in Figure 1). To enhance model interpretability, we adopted the Shapley Additive exPlanations (SHAP) method. SHAP uses Shapley values to quantify each feature’s contribution, enabling both global and individual-level explanations. The SHAP summary plot was used to aggregate the local impact of each feature across all instances and to visually present a comprehensive overview of global feature importance. Features were ranked vertically based on their mean absolute SHAP value, which instantly highlighted the most influential predictors.

The discriminative ability of the final model was evaluated using receiver operating characteristic (ROC) curves and the area under the curve (AUC), with AUC > 0.7 indicating acceptable discrimination and AUC > 0.8 indicating excellent discrimination. Calibration plots (CP) were generated to assess model calibration, and decision curve analysis (DCA) was performed for both the train and test sets to evaluate the clinical efficacy of the prediction model. The detailed methodological workflow is presented in the flow chat (Figure 1).

### 2.5 Statistical analysis

Continuous variables were expressed as mean ± standard deviation (SD) for normally distributed data or median (interquartile range, IQR) for skewed data, and compared using the independent t-test or Mann–Whitney U test, respectively. Categorical variables were coded as 1 (“yes”) or 0 (“no”) and summarized as frequencies and percentages, with group comparisons performed using the chi-square or Fisher’s exact test. Statistical significance was defined as a two-sided *p* < 0.05. All analyses were conducted in R (version 4.5). Feature selection was performed using Lasso via the “glmnet” packages. Machine Learning models were trained using the “caret” package, with hyperparameters optimized by standard grid search. The Shapiro–Wilk test was applied to assess the normality of continuous variables. Four machine learning algorithms were implemented. For light gradient boosting machine (LightGBM), hyperparameters included a learning rate of 0.1, 31 leaves per tree, L1/L2 regularization terms of 1, and a minimum of 20 samples per leaf; training employed 5-fold cross-validation with early stopping. The categorical boosting CatBoost model was tuned to a depth of 4, a learning rate of 0.01, 100 iterations, and an L2 regularization value of 0.1, utilizing a feature sampling rate (rsm) of 0.95 and 64 bit borders. Support vector machines (SVM) were optimized with a radial kernel (sigma = 0.134) and a cost parameter of 0.25. Finally, a K-nearest neighbors (KNN) model with k = 11 was fitted.

## 3 Result

### 3.1 Characteristics of EUGR and non-EUGR infants

This retrospective cohort study enrolled 288 very preterm patients divided into an EUGR and non-EUGR group. According to the exclusion criteria, 5 infants with congenital structural anomalies were excluded, along with 7 infants who died or were transferred, 3 infants with genetic or metabolic disorders, and 13 infants with incomplete data. A total of 260 preterm infants were included in this study. Based on the diagnostic criteria for EUGR, the cohort was stratified into two groups: the non-EUGR group (*n* = 90, 34.6%) and the EUGR group (*n* = 170, 65.4%). Clinical characteristics of infants with and without EUGR were presented in Table 1. Infants in the EUGR group had a significantly lower gestational age (29.35 weeks vs. 30.95 weeks, *p* < 0.001) and lower birth weight (1.23 kg vs. 1.38 kg, *p* < 0.001) compared to the non-EUGR group. Although the weight Z-scores (WZS) at birth and on day 7 and 14 showed no significant differences, the WZS at discharge was markedly lower in the EUGR group (−1.95 vs. −1.08, *p* < 0.001). Furthermore, the length of hospital stay was considerably longer for infants with EUGR (53 days vs. 35 days, *p* < 0.001). Compared with infants without EUGR, those with EUGR were more likely to fail to regain their birth weight within the first 14 days of life (11.18% vs. 2.22%, *p* = 0.012). Maternal GDM and HDP were significantly more prevalent among infants who developed EUGR (GDM: 42.94% vs. 20.00%, *p* < 0.001; HDP: 30.59% vs. 16.67%, *p* = 0.015). Infants in the EUGR group demonstrated a higher burden of specific comorbidities, marked by significantly elevated rates of anemia (76.47% vs. 61.11%, *p* = 0.009) and ROP (24.71% vs. 13.33%, *p* = 0.032). Furthermore, infants with EUGR had significantly lower BUN levels on postnatal day 14, with a smaller proportion reaching ≥ 3.2 mmol/L compared to those without EUGR (22.94% vs. 42.22%, *p* = 0.001).

**Table 1:** Demographic and clinical characteristics.

Variables	Total (*n* = 260)	Non-EUGR (*n* = 90)	EUGR (*n* = 170)	Statistic t/z/χ^2^	P*
Infants characteristics					
Male Sex, *n* (%)	158 (60.77)	60 (66.67)	98 (57.65)	χ^2^ = 2.01	0.156
Gestational age (w)	29.70 (28.70, 31.00)	30.95 (29.02, 31.67)	29.35 (28.60, 30.55)	Z = -4.50	<0.001
BW (kg)	1.31 (1.05, 1.46)	1.38 (1.17, 1.48)	1.23 (1.00, 1.42)	Z = -3.65	<0.001
Apgar-5 min score	10.00 (10.00, 10.00)	10.00 (10.00, 10.00)	10.00 (10.00, 10.00)	Z = -1.08	0.279
Weight on day 7 (kg)	1.26 (1.01, 1.40)	1.34 (1.16, 1.44)	1.20 (0.98, 1.37)	Z = -4.04	<0.001
Weight on day 14 (kg)	1.44 (1.14, 1.60)	1.54 (1.36, 1.68)	1.33 (1.08, 1.55)	Z = -4.74	<0.001
WZS at born	−0.32 ± 0.72	−0.39 ± 0.66	−0.28 ± 0.75	t = −1.21	0.226
WZS-7	−0.94 (−1.37, −0.46)	−0.88 (−1.46, −0.47)	−0.95 (−1.33, −0.45)	Z = -0.46	0.646
WZS-14	−1.12 ± 0.65	−1.09 ± 0.61	−1.13 ± 0.68	t = 0.47	0.641
WZS at discharge	−1.54 (−2.32, −0.96)	−1.08 (−1.62, −0.48)	−1.95 (−2.79, −1.23)	Z = -7.20	<0.001
Growth velocity of weight (g/kg/d)	16.49 (11.14, 21.77)	18.39 (14.35, 22.66)	15.44 (9.81, 20.72)	Z = -2.79	0.005
Length of Stay (d)	48.00 (35.75, 68.00)	35.00 (27.25, 52.00)	53.00 (41.00, 70.00)	Z = -6.00	<0.001
Multiple birth, *n* (%)	80 (30.77)	23 (25.56)	57 (33.53)	χ^2^ = 1.76	0.185
Delivery, *n* (%)	157 (60.38)	55 (61.11)	102 (60.00)	χ^2^ = 0.03	0.862
SGA, *n* (%)	29 (11.15)	9(10.00)	20(11.76)	χ^2^ = 0.05	0.824
Maternal characteristics					
Maternal age (year)	32.00 (28.00, 35.00)	31.00 (28.00, 34.00)	32.00 (28.00, 35.00)	Z = -0.80	0.424
GDM, *n* (%)	91 (35.00)	18 (20.00)	73 (42.94)	χ^2^ = 13.61	<0.001
HDP, *n* (%)	67 (25.77)	15 (16.67)	52 (30.59)	χ^2^ = 5.96	0.015
ART, *n* (%)	60 (23.08)	18 (20.00)	42 (24.71)	χ^2^ = 0.73	0.392
CAM, *n* (%)	58 (22.31)	17 (18.89)	41 (24.12)	χ^2^ = 0.93	0.335
Placental abruption, n (%)	27 (10.38)	7 (7.78)	20 (11.76)	χ^2^ = 1.01	0.316
PROM>18 h, *n* (%)	77 (29.62)	24 (26.67)	53 (31.18)	χ^2^ = 0.57	0.449
IUGR, *n* (%)	38 (14.62)	16 (17.78)	22 (12.94)	χ^2^ = 1.10	0.294
Nutritional factors					
Milk intake on day 7 (mL/kg),	18.40 (6.80, 37.20)	19.50 (6.75, 35.95)	18.30 (7.30, 37.50)	Z = -0.50	0.614
Milk intake on day 14 (mL/kg),	57.10 (30.12, 86.30)	57.10 (25.52, 91.30)	57.35 (31.85, 84.35)	Z = -0.39	0.698
The duration of mechanical ventilation (d)	5.00 (1.00, 8.00)	5.00 (1.00, 7.00)	6.00 (1.00, 8.75)	Z = -1.41	0.159
Time to initiation of Feeding (d)	3.00 (2.00, 4.00)	3.00 (2.00, 4.00)	3.00 (2.00, 4.75)	Z = -0.76	0.449
Amino acid in TPN on day 7 (g/d)	3.33 (2.77, 3.58)	3.21 (2.58, 3.51)	3.40 (2.85, 3.58)	Z = -1.89	0.059
Amino acid in TPN on day 14 (g/d)	2.87 (2.48, 3.03)	2.86 (2.46, 3.00)	2.88 (2.48, 3.04)	Z = -0.83	0.407
Lipid emulsion in TPN on day 7 (g/d)	2.75 (2.08, 3.30)	2.64 (1.95, 3.23)	2.77 (2.21, 3.31)	Z = -1.37	0.172
Lipid emulsion on day 14 (g/d)	2.77 (2.18, 2.99)	2.73 (1.99, 2.99)	2.77 (2.35, 2.99)	Z = -1.01	0.311
Cumulative duration of fasting during the initial 14 days (d)	3.00 (2.00, 5.00)	3.00 (2.00, 6.00)	3.00 (2.00, 5.00)	Z = -0.24	0.810
Time to regain birth weight	8.00 (6.00, 11.00)	8.00 (6.00, 11.00)	8.50 (7.00, 10.00)	Z = -0.06	0.950
Time to regain birth weight within 14 days *n* (%)				χ^2^ = 6.35	0.012
>14d	21 (8.08)	2 (2.22)	19 (11.18)		
≤14d	239 (91.92)	88 (97.78)	151 (88.82)		
Laboratory variables					
CR-14(μmol/l)	43.20 (36.68, 51.20)	42.55 (34.02, 48.38)	43.40 (37.15, 52.82)	Z = -1.91	0.057
GLU-14(mmol/l)	5.10 (4.32, 5.85)	5.00 (4.38, 5.80)	5.15 (4.27, 5.93)	Z = -0.43	0.664
BUN-14(mmol/l)	2.22 (1.48, 3.22)	2.09 (1.44, 3.25)	2.3 (1.5, 3.2)	Z = -0.54	0.591
BUN *n* (%)				χ^2^ = 10.50	0.001
<3.2 mmol/L	183 (70.38)	52 (57.78)	131 (77.06)		
≥3.2 mmol/L	77 (29.62)	38 (42.22)	39 (22.94)		
HGB-14 (g/L)	124.00 (97.75, 144.25)	124.00 (93.25, 148.00)	123.50 (100.25, 142.00)	Z = -0.17	0.866
Morbidities before 14 days of life					
NRDS, *n* (%)	111 (42.69)	32 (35.56)	79 (46.47)	χ^2^ = 2.87	0.090
PDA, *n* (%)	82 (31.54)	26 (28.89)	56 (32.94)	χ^2^ = 0.45	0.504
EOS, *n* (%)	24 (9.23)	5 (5.56)	19 (11.18)	χ^2^ = 2.22	0.136
PH, *n* (%)	16 (6.15)	5 (5.56)	11 (6.47)	χ^2^ = 0.09	0.770
IVH, *n* (%)	152 (58.46)	54 (60.00)	98 (57.65)	χ^2^ = 0.13	0.714
Morbidities after 14 days of life					
ROP, *n* (%)	54 (20.77)	12 (13.33)	42 (24.71)	χ^2^ = 4.63	0.032
Anemia, *n* (%)	185 (71.15)	55 (61.11)	130 (76.47)	χ^2^ = 6.76	0.009
Neonatal cholestasis, *n* (%)	14 (5.41)	5 (5.62)	9 (5.29)	χ^2^ = 0.00	1.000
LOS, *n* (%)	23 (8.85)	8 (8.89)	15 (8.82)	χ^2^ = 0.00	0.986
NEC, *n* (%)	37 (14.23)	10 (11.11)	27 (15.88)	χ^2^ = 1.10	0.295
BPD, *n* (%)	53 (20.38)	22 (24.44)	31 (18.24)	χ^2^ = 1.40	0.237

WZS, weight Z-score; WZS-7, weight Z score on day 7; WZS-14, weight Z score on day 14; WZS at discharge; weight Z score at discharge; BW, birth weight; GA, gestational age; CR-14 creatinine on day 14; Glu-14,glucose on day 14; BUN-14, blood urea nitrogen on day 14; HGB-14, hemoglobin on day 14; GDM, gestational diabetes Mellitus HDP, hypertensive disorders of pregnancy; SGA, small for gestational age. ART, assisted reproductive technology; NRDS, neonatal respiratory distress syndrome; PDA patent ductus arteriosus; EOS, early-onset sepsis. PPHN, persistent pulmonary hypertension; CAM, chorioamnionitis; PROM, premature rupture of membranes. IVH, intraventricular hemorrhage; IUGR, intrauterine growth restriction; ROP retinopathy of prematurity. LOS, late-onset sepsis; NEC, necrotizing enterocolitis; BPD, bronchopulmonary dysplasia.

t: *t*-test, Z: Mann–Whitney test, χ^2^: Chi-square test, numbers represent mean ± SD for parametric continuous variables, median (IQR) for non-parametric continuous variables and n (%) for categorical variables. P*-values obtained from independent samples t-test or Mann–Whitney non-parametric test, used to compare continuous variables between EUGR group and non-EUGR group.

### 3.2 Comparison of baseline characteristics between the train and test sets

The dataset was randomly split into a train and test cohort with a ratio of 7:3. All the compared parameters were comparable between train and test cohort (*p* > 0.05). Baseline characteristics of train and test group were presented in Table 2.

**Table 2:** Comparison of baseline characteristics between the Train and Test sets.

Variables	Total (*n* = 260)	Test set (*n* = 78)	Train set (*n* = 182)	Statistic t/Z/χ^2^	P*
Infants characteristics					
Male sex, *n* (%)	158 (60.77)	48 (61.54)	110 (60.44)	χ^2^ = 0.03	0.868
Gestational age (w)	29.70 (28.70, 31.00)	29.40 (28.63, 31.10)	29.90 (28.75, 31.00)	Z = −0.79	0.432
BW (kg)	1.31 (1.05, 1.46)	1.23 (1.04, 1.40)	1.32 (1.05, 1.47)	Z = −1.19	0.234
Apgar-5 min score	10.00 (10.00, 10.00)	10.00 (10.00, 10.00)	10.00 (10.00, 10.00)	Z = −0.20	0.839
Weight on day 7 (kg)	1.26 (1.01, 1.40)	1.20 (1.00, 1.38)	1.29 (1.03, 1.41)	Z = −1.39	0.166
Weight on day 14 (kg)	1.44 (1.14, 1.60)	1.38 (1.13, 1.56)	1.46 (1.15, 1.61)	Z = −1.16	0.248
WZS at born	−0.32 ± 0.72	−0.37 ± 0.73	−0.30 ± 0.72	t = −0.71	0.478
WZS-7	−0.94 (−1.37, −0.46)	−0.95 (−1.47, −0.49)	−0.94 (−1.33, −0.45)	Z = −0.65	0.515
WZS-14	−1.12 ± 0.65	−1.15 ± 0.64	−1.10 ± 0.66	t = −0.49	0.622
Birth weight (kg)	1.31 (1.05, 1.46)	1.23 (1.04, 1.40)	1.32 (1.05, 1.47)	Z = −1.19	0.234
Growth velocity of weight (g/kg/d)	16.49 (11.14, 21.77)	16.18 (10.59, 21.97)	16.56 (11.18, 21.65)	Z = -0.31	0.754
Apgar-5 min score	10.00 (10.00, 10.00)	10.00 (10.00, 10.00)	10.00 (10.00, 10.00)	Z = −0.20	0.839
Twin, *n* (%)	80 (30.77)	24 (30.77)	56 (30.77)	χ^2^ = 0.00	1.000
Cesarean delivery, *n* (%)	157 (60.38)	46 (58.97)	111 (60.99)	χ^2^ = 0.09	0.761
SGA, *n* (%)	29(11.15)	12(15.38)	17(9.34)	χ^2^ = 1.45	0.229
Maternal characteristics					
Maternal age (year)	32.00 (28.00, 35.00)	32.00 (28.00, 35.00)	32.00 (28.00, 35.00)	Z = −0.80	0.424
GDM, *n* (%)	91 (35.00)	29 (37.18)	62 (34.07)	χ^2^ = 0.23	0.630
HDP, *n* (%)	67 (25.77)	21 (26.92)	46 (25.27)	χ^2^ = 0.08	0.781
ART, *n* (%)	60 (23.08)	23 (29.49)	37 (20.33)	χ^2^ = 2.58	0.108
CAM, *n* (%)	58 (22.31)	18 (23.08)	40 (21.98)	χ^2^ = 0.04	0.845
Placental abruption, *n* (%)	27 (10.38)	6 (7.69)	21 (11.54)	χ^2^ = 0.39	0.534
PROM >18 h, *n* (%)	77 (29.62)	21 (26.92)	56 (30.77)	χ^2^ = 0.57	0.449
IUGR, *n* (%)	38 (14.62)	7 (8.97)	31 (17.03)	χ^2^ = 2.84	0.092
Nutritional variables					
Milk intake on day 7 (mL/kg)	57.10 (30.12, 86.30)	56.10 (32.23, 89.60)	58.90 (29.68, 84.42)	Z = −0.06	0.953
Milk intake on day 14 (mL/kg)	57.10 (30.12, 86.30)	57.10 (25.52, 91.30)	57.35 (31.85, 84.35)	Z = −0.39	0.698
The duration of mechanical ventilation (d)	5.00 (1.00, 8.00)	5.00 (1.00, 7.00)	6.00 (1.00, 8.75)	Z = −1.41	0.159
Time to initiation of Feeding (d)	3.00 (2.00, 4.00)	3.00 (2.00, 4.00)	3.00 (2.00, 4.00)	Z = −1.03	0.305
Amino acid in TPN on day 7 (g/d)	3.33 (2.77, 3.58)	3.28 (2.79, 3.56)	3.35 (2.76, 3.58)	Z = −0.42	0.672
Amino acid in TPN on day 14 (g/d)	2.87 (2.48, 3.03)	2.86 (2.47, 3.02)	2.88 (2.48, 3.03)	Z = −0.22	0.823
Lipid emulsion in TPN on day 7 (g/d)	2.75 (2.08, 3.30)	2.75 (2.19, 3.28)	2.75 (2.08, 3.30)	Z = −0.17	0.861
Lipid emulsion in TPN on day 14 (g/d)	2.77 (2.18, 2.99)	2.75 (2.23, 2.96)	2.79 (2.17, 3.00)	Z = −0.57	0.566
Days to cumulative duration of fasting during the initial 14 days (d)	3.00 (2.00, 5.00)	3.00 (2.00, 6.00)	3.00 (2.00, 5.00)	Z = −0.36	0.720
Time to regain birth weight, *n* (%)				χ^2^ = 0.12	0.728
>14d	21 (8.08)	7 (8.97)	14 (7.69)		
≤14d	239 (91.92)	71 (91.03)	168 (92.31)		
Laboratory variables					
CR-14(umol/l)	43.20 (36.68, 51.20)	42.55 (34.02, 48.38)	43.40 (37.15, 52.82)	Z = −1.91	0.057
GLU-14(mmol/l)	5.10 (4.32, 5.85)	5.00 (4.38, 5.80)	5.15 (4.27, 5.93)	Z = −0.43	0.664
BUN-14, *n* (%)				χ^2^ = 0.32	0.573
<3.2 mmol/L	183 (70.38)	53 (67.95)	130 (71.43)		
≥3.2 mmol/L	77 (29.62)	25 (32.05)	52 (28.57)		
HGB-14(g/L)	128.00 (114.75, 148.00)	125.50 (115.00, 143.00)	129.50 (114.25, 148.00)	Z = −0.89	0.374
Morbidities before 14 days of life					
NRDS, *n* (%)	111 (42.69)	36 (46.15)	75 (41.21)	χ^2^ = 0.55	0.460
PDA, *n* (%)	82 (31.54)	30 (38.46)	52 (28.57)	χ^2^ = 2.47	0.116
EOS, *n* (%)	24 (9.23)	9 (11.54)	15 (8.24)	χ^2^ = 0.71	0.400
PPHN, *n* (%)	16 (6.15)	5 (6.41)	11 (6.04)	χ^2^ = 0.00	1.000
IVH, *n* (%)	152 (58.46)	49 (62.82)	103 (56.59)	χ^2^ = 0.87	0.350

WZS, weight Z-score; WZS-7,weight Z score on day 7; WZS-14,weight Z score on day 14; BW, birth weight; GA, gestational age; CR-14,creatinine on day 14; Glu-14,glucose on day 14; BUN-14, blood urea nitrogen on day 14; HGB-14, hemoglobin on day 14; GDM, gestational diabetes Mellitus HDP, hypertensive disorders of pregnancy; SGA, small for gestational age; ART, assisted reproductive technology; NRDS, neonatal respiratory distress syndrome; PDA patent ductus arteriosus.; EOS, early-onset sepsis; PPHN, persistent pulmonary hypertension; CAM, chorioamnionitis; PROM, premature rupture of membranes; IVH, intraventricular hemorrhage; IUGR, intrauterine growth restriction.

t, *t*-test, Z: Mann–Whitney test, χ^2^: Chi-square test, Numbers represent mean ± SD for parametric continuous variables, median (IQR) for non-parametric continuous variables and *n* (%) for categorical variables. P*-values obtained from independent samples t-test or Mann–Whitney non-parametric test, used to compare variables between train set and test set.

### 3.3 Univariate logistic regression analysis of the train cohort

Univariable logistic regression was performed for all candidate predictors in the train cohort. For infants who developed longitudinal EUGR, GDM (OR = 2.98, *p* = 0.003) and HDP (OR = 2.52, *p* = 0.020) were identified as significant risk factors. In contrast, time to regain birth weight of <14 days (OR = 0.12, *p* = 0.045) and BUN ≥3.2 mmol/L (OR = 0.44, *p* = 0.016), higher birth weight (OR = 0.13, *p* = 0.002), gestational age (OR = 0.62, *p* < 0.001), weight on day 7 (OR = 0.07, *p* < 0.001) and day 14 (OR = 0.08, *p* < 0.001) demonstrated protective effects (shown in Table 3). The remaining variables did not reach statistical significance in the univariable analysis (*p* > 0.05).

**Table 3:** Results of univariate logistic regression analysis in the train set.

Variables	β	S.E	Z	P*	OR (95%CI)
Sex, male	−0.63	0.33	−1.92	**0.055**	0.54 (0.28 ~ 1.01)
Time to regain birth weight ≤14 d	−2.10	1.05	−2.00	**0.045**	0.12 (0.02 ~ 0.95)
GDM	1.09	0.36	3.02	**0.003**	2.98 (1.47 ~ 6.06)
HDP	0.92	0.40	2.32	**0.020**	2.52 (1.16 ~ 5.49)
SGA	0.67	0.59	1.13	0.259	1.96 (0.61 ~ 6.27)
BUN ≥3.2 mmol/L	−0.81	0.34	−2.41	**0.016**	0.44 (0.23 ~ 0.86)
ART	0.37	0.40	0.92	0.356	1.44 (0.66 ~ 3.15)
NRDS	0.52	0.32	1.62	0.105	1.68 (0.90 ~ 3.16)
PDA	0.22	0.35	0.63	0.527	1.25 (0.63 ~ 2.46)
EOS	0.48	0.61	0.80	0.423	1.62 (0.50 ~ 5.32)
CAM	0.67	0.40	1.66	**0.097**	1.95 (0.89 ~ 4.31)
PPHN	−0.00	0.65	−0.01	0.994	1.00 (0.28 ~ 3.54)
PROM > 18 h	0.50	0.35	1.43	0.152	1.64 (0.83 ~ 3.25)
Placental abruption	0.15	0.49	0.30	0.767	1.16 (0.44 ~ 3.03)
Twin	0.26	0.34	0.77	0.441	1.30 (0.67 ~ 2.53)
Cesarean delivery	−0.18	0.32	−0.55	0.581	0.84 (0.45 ~ 1.57)
IVH	−0.16	0.31	−0.51	0.608	0.85 (0.46 ~ 1.57)
IUGR	−0.13	0.41	−0.31	0.756	0.88 (0.40 ~ 1.95)
BW (kg)	−2.05	0.66	−3.12	**0.002**	0.13 (0.04 ~ 0.47)
GA (w)	−0.47	0.13	−3.64	**<0.001**	0.62 (0.48 ~ 0.80)
WZ-score at born	0.12	0.22	0.57	0.570	1.13 (0.74 ~ 1.73)
Milk intake on day 7 (ml)	−0.00	0.01	−0.28	0.780	1.00 (0.98 ~ 1.01)
Milk intake on day 14 (ml)	0.00	0.00	0.06	0.953	1.00 (0.99 ~ 1.01)
The duration of mechanical ventilation (d)	0.04	0.03	1.07	0.285	1.04 (0.97 ~ 1.11)
CR-14 (μmol/L)	0.00	0.01	0.34	0.736	1.00 (0.98 ~ 1.03)
GLU-14 (mmol/L)	−0.04	0.10	−0.36	0.719	0.96 (0.79 ~ 1.17)
HGB-14 (g/L)	−0.00	0.00	−0.49	0.622	1.00 (0.99 ~ 1.00)
Amino acid in TPN on day 7 (g/kg/d)	0.31	0.24	1.30	0.194	1.36 (0.86 ~ 2.15)
Amino acid in TPN on day 14 (g/kg/d)	0.17	0.25	0.66	0.511	1.18 (0.72 ~ 1.93)
Lipid emulsion in TPN on day 7 (g/d)	0.12	0.19	0.64	0.525	1.13 (0.78 ~ 1.62)
Lipid emulsion in TPN on day 14 (g/d)	0.14	0.22	0.62	0.532	1.15 (0.74 ~ 1.77)
Cumulative duration of fasting during the initial 14 days (d)	−0.06	0.07	−0.92	0.356	0.94 (0.82 ~ 1.07)
Weight on day 7 (kg)	−2.61	0.72	−3.63	**<0.001**	0.07 (0.02 ~ 0.30)
Weight on day 14 (kg)	−2.53	0.63	−4.00	**<0.001**	0.08 (0.02 ~ 0.28)
WZS-7	0.01	0.26	0.02	0.981	1.01 (0.60 ~ 1.69)
WZS-14	−0.22	0.24	−0.94	0.350	0.80 (0.51 ~ 1.27)
Time to initiation of Feeding (d)	−0.01	0.07	−0.20	0.842	0.99 (0.86 ~ 1.13)
Mother age (year)	0.01	0.03	0.36	0.718	1.01 (0.95 ~ 1.07)
Growth Velocity (g/kg/d)	−0.02	0.01	−1.47	0.140	0.98 (0.96 ~ 1.01)

WZS, weight Z-score; WZS-7, weight Z score on day 7; WZS-14, weight Z score on day 14; BW, birth weight; GA, gestational age; CR-14, creatinine on day 14; Glu-14, glucose on day 14; BUN-14, blood urea nitrogen on day 14; HGB-14, hemoglobin on day 14; GDM, gestational diabetes Mellitus; HDP; hypertensive disorders of pregnancy; SGA, small for gestational age; ART, assisted reproductive technology; PPHN, persistent pulmonary hypertension; NRDS, neonatal respiratory distress syndrome. PDA, patent ductus arteriosus. EOS, early-onset sepsis. CAM, chorioamnionitis; PROM, premature rupture of membranes. IVH, intraventricular hemorrhage; IUGR: intrauterine growth restriction. Prematurity variables with *p* < 0.1 are bolded in the table. OR: Odds Ratio, CI: Confidence Interval.

### 3.4 Feature selection: LASSO

**Figure 2:**
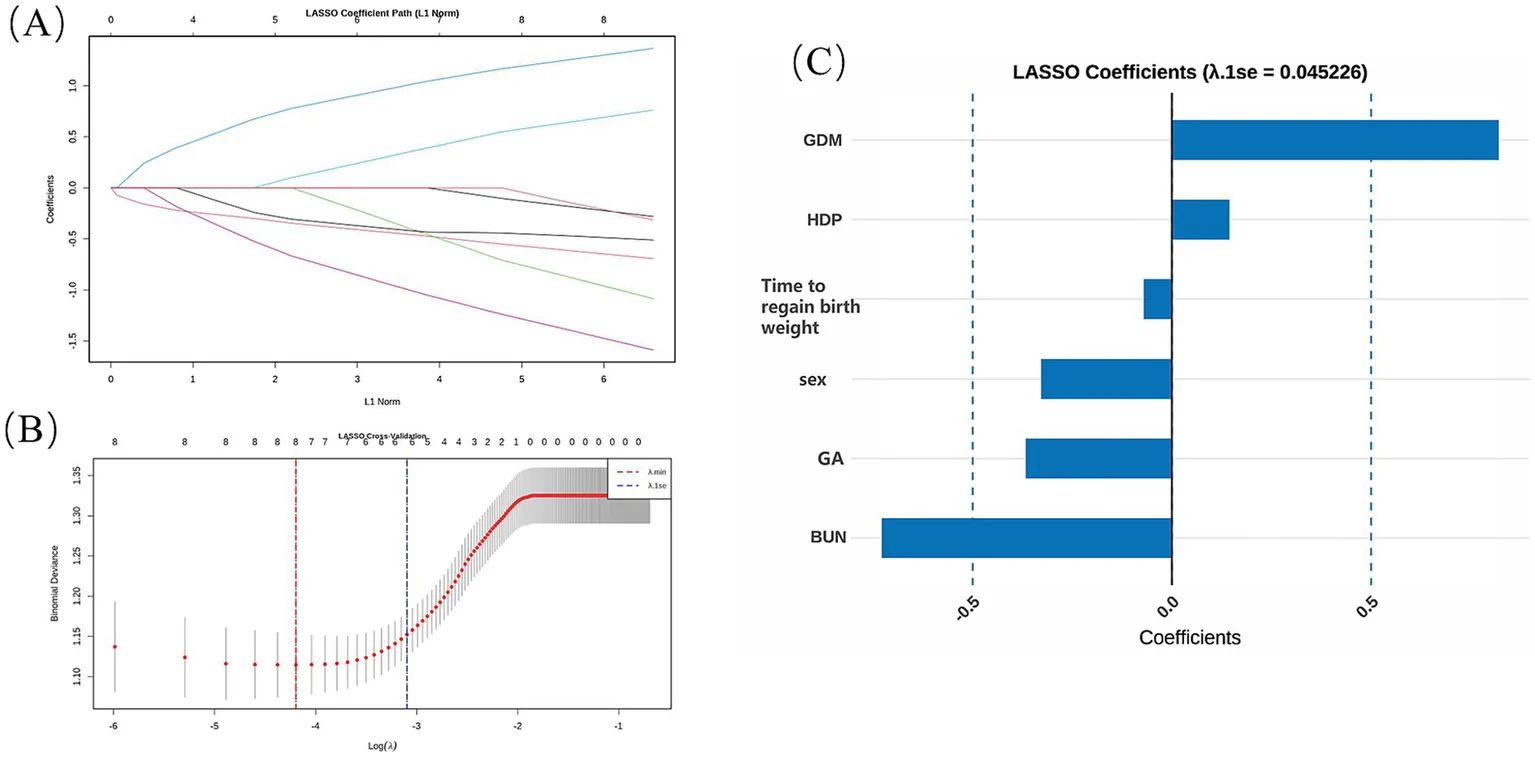
**(A)** Relationship between the L1-norm and coefficients in the LASSO regression model. The L1-norm acts as the regularization term in LASSO. **(B)** Association between the *lambda* (*λ*) value and binomial deviance. The vertical dashed lines indicate the *lambda* value corresponding to the minimum mean squared error (left) and the value one standard error above it (right). **(C)** Horizontal bar plot showing the coefficients of variables selected by the LASSO model. Bar length and direction correspond to the absolute value and sign of each coefficient, respectively, reflecting the strength and direction of association with the outcome. BUN, blood urea nitrogen on day 14; GA, gestational age; GDM, gestational diabetes mellitus; HDP, hypertensive disorders of pregnancy; NRDS, neonatal respiratory distress syndrome; PROM, premature rupture of membranes; W-7, weight on day 7 (kg), W-14, weight on day 14 (kg).

Based on the results of the univariate logistic regression analysis in the train cohort, 10 variables with *p* < 0.1 (shown in Table 3) were further included in the LASSO regression for variable selection. In the train cohort, the coefficient path plot and the 10-fold cross-validation curve of the LASSO regression analysis were shown in Figures 2A,B. To achieve optimal model performance, the *λ* min was selected after 10-fold cross-validation. The variables retained by the LASSO analysis included sex, GA, GDM, HDP, BUN, time to regain birth weight (Figure 2C), incorporated the above six variables into each model for further construction and validation.

### 3.5 Evaluation of models

**Figure 3:**
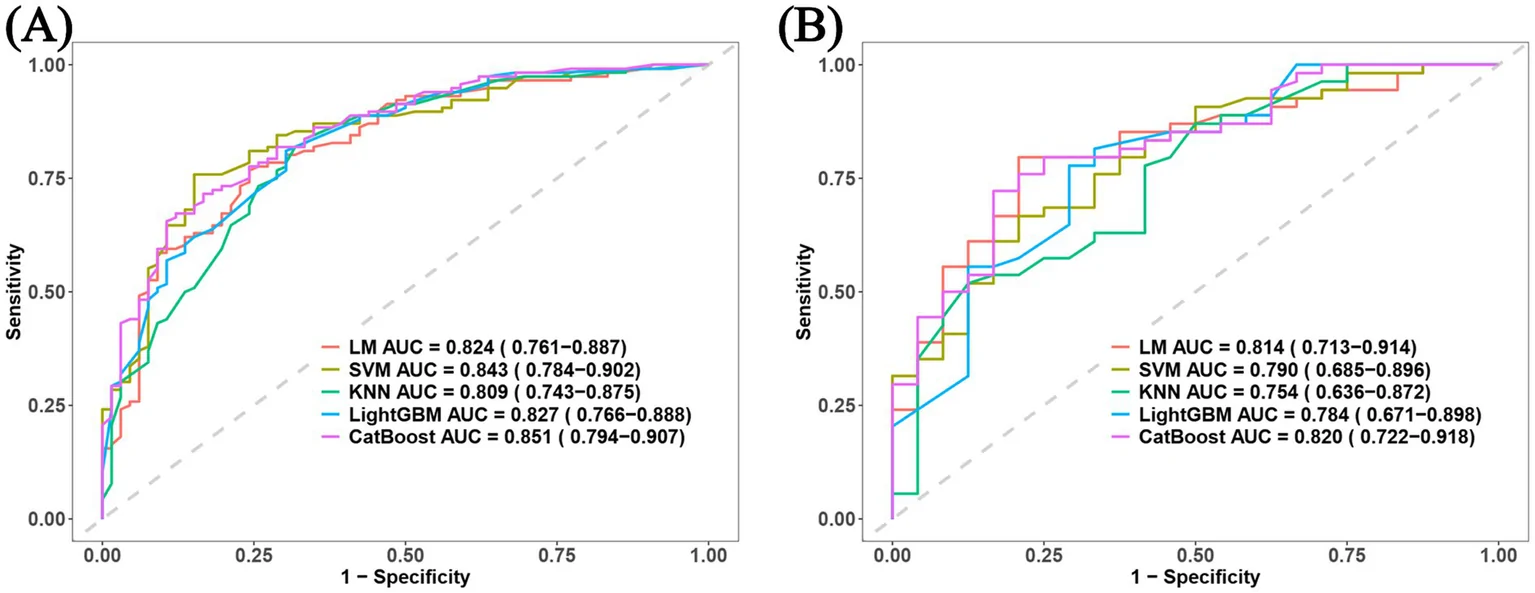
Receiver operating characteristic (ROC) curves and corresponding area under the curve (AUC) values with 95% confidence intervals for each model, based on the train dataset **(A)** and test dataset **(B)**. LM, logistic regression model; SVM, support vector machine; KNN, *K*-nearest neighbors; LightGBM, light gradient boosting machine; CatBoost, categorical boosting.

Four machine learning models and logistic regression model were developed to predict the occurrence of EUGR. Figure 3 presented the discriminative performance of these four models and the logistic regression model based on the AUC of the train and test set. Among these models, the CatBoost model achieved the highest AUC in the test cohort (AUC = 0.820), outperforming logistic regression (AUC = 0.814), support vector machine (AUC = 0.790), LightGBM (AUC = 0.784), and K-nearest neighbors (AUC = 0.754), indicating predictive performance and deployment suitability. To further compare the discrimination performance of machine learning models with the logistic regression model, the evaluation metrics of each model were presented in Table 4 and Supplementary Figure 1. Logistic regression and CatBoost model achieved the best performance, with no statistically significant difference observed between AUC (shown in Supplementary Table 1). Logistic regression model demonstrated superior overall parameter performance compared to CatBoost and other predictive models. Furthermore, its metrics remained stable from the training set to the validation set. Therefore, logistic regression was ultimately selected as the optimal prediction model.

**Table 4:** Evaluation metrics of machine learning algorithms and logistic regression model.

Model	Data	AUC	Accuracy	Sensitivity	Specificity	Precision	F1-score
LM	Train	0.824	0.764	0.767	0.758	0.848	0.805
	Test	0.814	0.782	0.778	0.792	0.894	0.832
LightGBM	Train	0.827	0.769	0.810	0.697	0.825	0.817
	Test	0.784	0.718	0.685	0.792	0.843	0.819
CatBoost	Train	0.851	0.747	0.672	0.879	0.907	0.772
	Test	0.820	0.628	0.537	0.833	0.879	0.667
SVM	Train	0.843	0.791	0.759	0.849	0.898	0.822
	Test	0.790	0.692	0.648	0.876	0.876	0.744
KNN	Train	0.809	0.753	0.767	0.727	0.832	0.798
	Test	0.754	0.667	0.685	0.625	0.787	0.733

LM, logistic regression model; SVM, support vector machine; KNN, K-nearest neighbors; LightGBM, light gradient boosting machine.

### 3.6 Construction of the logistic regression model

Variables identified by the LASSO, including sex, GA, GDM, HDP, time to regain birth weight, BUN, were incorporated into a multivariable logistic regression model. The final multivariable analysis identified sex, GDM, HDP, GA, BUN, and time to regain birth weight as independent predictors of EUGR (shown in Table 5). The corresponding regression equation was as follows:

**Table 5:** Results of multivariable logistic regression analysis in the train set.

Variables	β	S.E	Z	*P*	OR (95%CI)
Intercept	28.89	5.58	5.18	<0.001	-
Sex-male	−0.82	0.41	−2.03	0.043	0.44 (0.20 ~ 0.97)
Time to regain birth weight	−2.38	1.11	−2.15	0.032	0.09 (0.01 ~ 0.81)
GDM	1.14	0.43	2.67	0.008	3.14 (1.35 ~ 7.28)
HDP	1.07	0.49	2.21	0.027	2.93 (1.13 ~ 7.59)
BUN-14	−1.77	0.46	−3.87	<0.001	0.17 (0.07 ~ 0.42)
GA	−0.85	0.18	−4.79	<0.001	0.43 (0.30 ~ 0.60)

BUN-14, blood urea nitrogen on day 14; GDM, gestational diabetes mellitus; HDP, hypertensive disorders of pregnancy; GA, gestational age.

log(P) = 28.8927–0.8215(X1)-2.3831(X2) + 1.1446(X3) + 1.0739(X4)-1.7694(X5)-0.8535(X6), where X1 represents sex, X2 represents time to regain birth weight, X3 represents gestational diabetes mellitus, X4 represents hypertensive disorders of pregnancy, X5 represents blood urea nitrogen, and X6 represents gestational age.

### 3.7 Validation and performance evaluation of the nomogram model

#### 3.7.1 Discrimination

**Figure 4:**
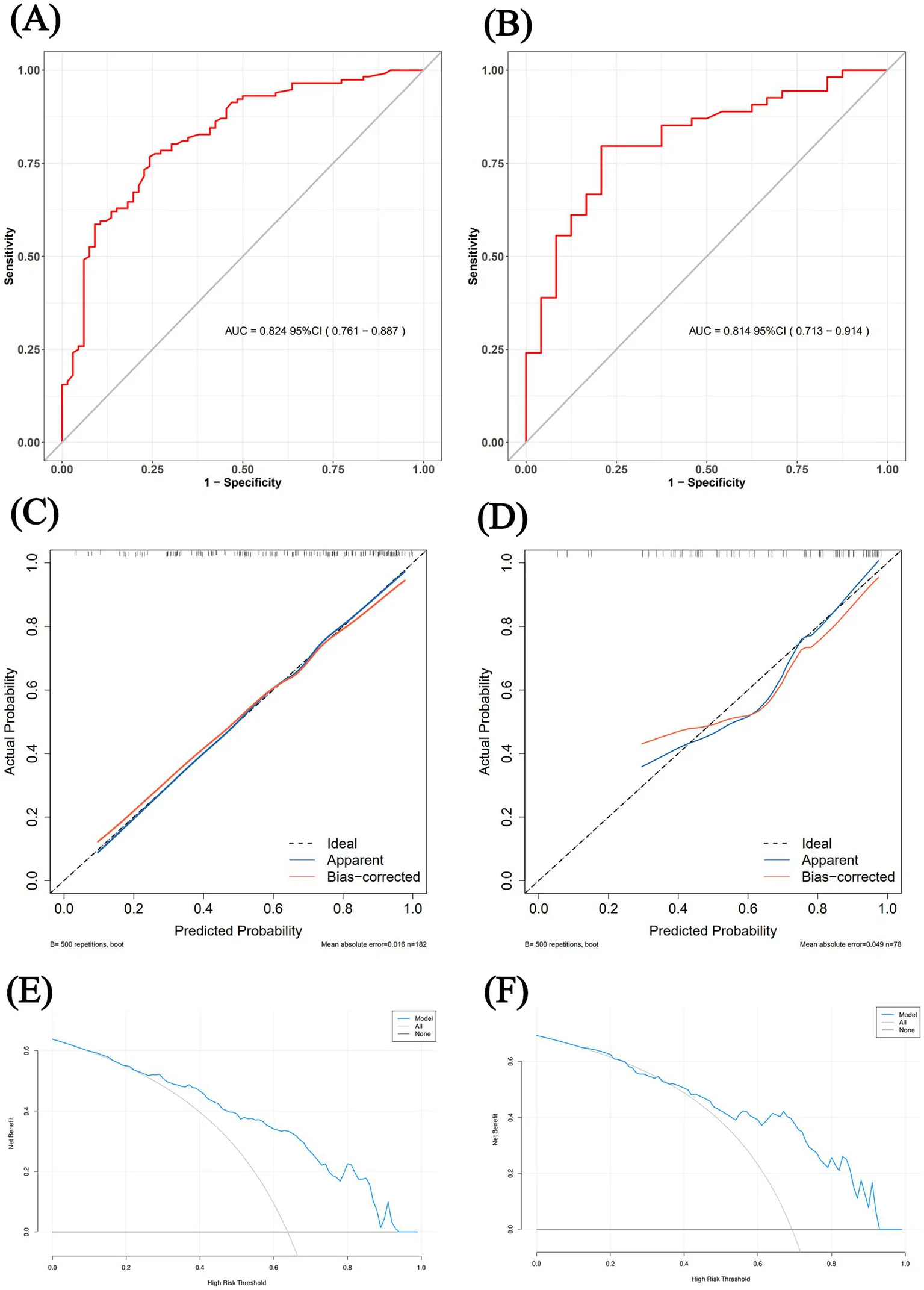
**(A)** ROC curves and AUC values for logistic regression on train sets. **(B)** ROC curves and AUC values for logistic regression on test sets. **(C)** Calibration plot of predicted probabilities vs. observed outcomes (train set). **(D)** Calibration plot of predicted probabilities vs. observed outcomes (test set). **(E)** Decision curve analysis (DCA) for the logistic regression model on the train set, depicting the net benefit across a range of threshold probabilities. **(F)** Decision curve analysis (DCA) for the logistic regression model on the test set, depicting the net benefit across a range of threshold probabilities.

ROC curves were generated for both the train and test cohorts based on the predicted probabilities derived from the logistic regression model. The AUC was 0.824 (95% CI: 0.761–0.887) in the train cohort and 0.814 (95% CI: 0.713–0.914) in the test cohort, indicating good discriminative performance of the nomogram (shown in Figures 4A,B).

#### 3.7.2 Calibration

Calibration plots were constructed using data from the train and test cohorts. The x-axis represented the predicted probability of EUGR, and the y-axis represented the observed incidence. The plots demonstrated close agreement between predicted and observed risks in both cohorts. Internal validation was performed using bootstrap resampling (500 iterations), and model calibration was further assessed using the Hosmer–Lemeshow test. The results showed X^2^ = 9.6275, *p* = 0.2921 for the train cohort and X^2^ = 6.2056, *p* = 0.6242 for the test cohort, indicating no significant deviation between predicted and observed outcomes (*p* > 0.05) and demonstrating good calibration (shown in Figures 4C,D).

#### 3.7.3 Decision curve

Decision curve analysis (DCA) was conducted for both the train and test cohorts to evaluate the clinical benefit of the predictive model. As shown in Figure 4, the threshold probability was displayed on the x-axis and the net benefit on the y-axis. When the threshold probability ranged from 25 to 95% in the train cohort and from 35 to 95% in the test cohort, the decision curve of the nomogram (blue line) provided a higher net benefit than the strategies of “treat all patients” (gray line) or “treat none” (black line). These results indicated that within these threshold ranges, applying the nomogram to guide clinical decision-making yielded additional net benefit and demonstrated good clinical applicability (Figures 4E,F).

#### 3.7.4 Nomogram construction

**Figure 5:**
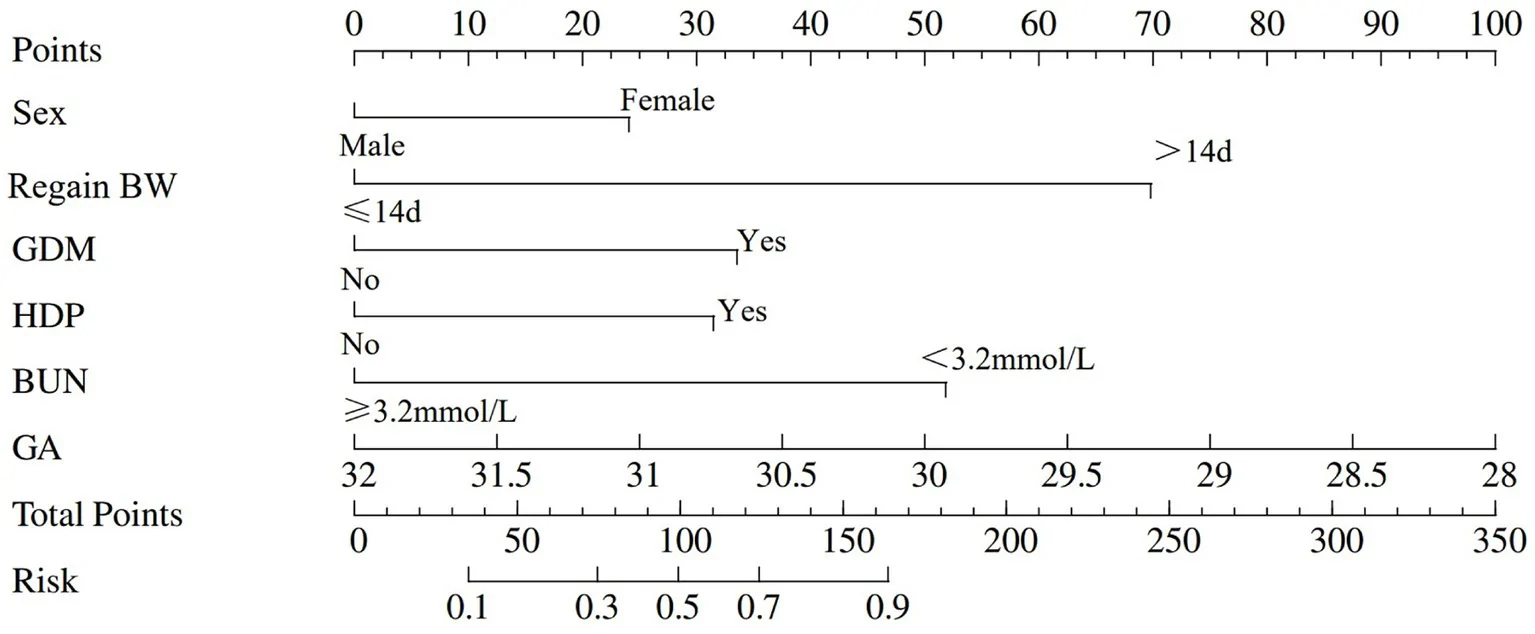
Nomogram of the logistic model. BUN, blood urea nitrogen on day 14, GDM, gestational diabetes mellitus, HDP, hypertensive disorders of pregnancy, GA, gestational age (W), Regain BW: Time to regain birth weight (d).

A nomogram was developed based on multivariable logistic regression to predict the risk of early EUGR in very preterm infants (28 to 31^+6^ weeks). The model incorporated six clinical factors: sex, GA, GDM, HDP, BUN on day 14, and time to regain birth weight. Points were assigned to each factor using the nomogram scale, and the total score corresponded to the predicted probability of longitudinal EUGR. A total score exceeding 100 points predicted a > 50% risk of EUGR at discharge, while a score above 165 points predicted a > 90% risk (shown in Figure 5).

#### 3.7.5 Model interpretability

**Figure 6:**
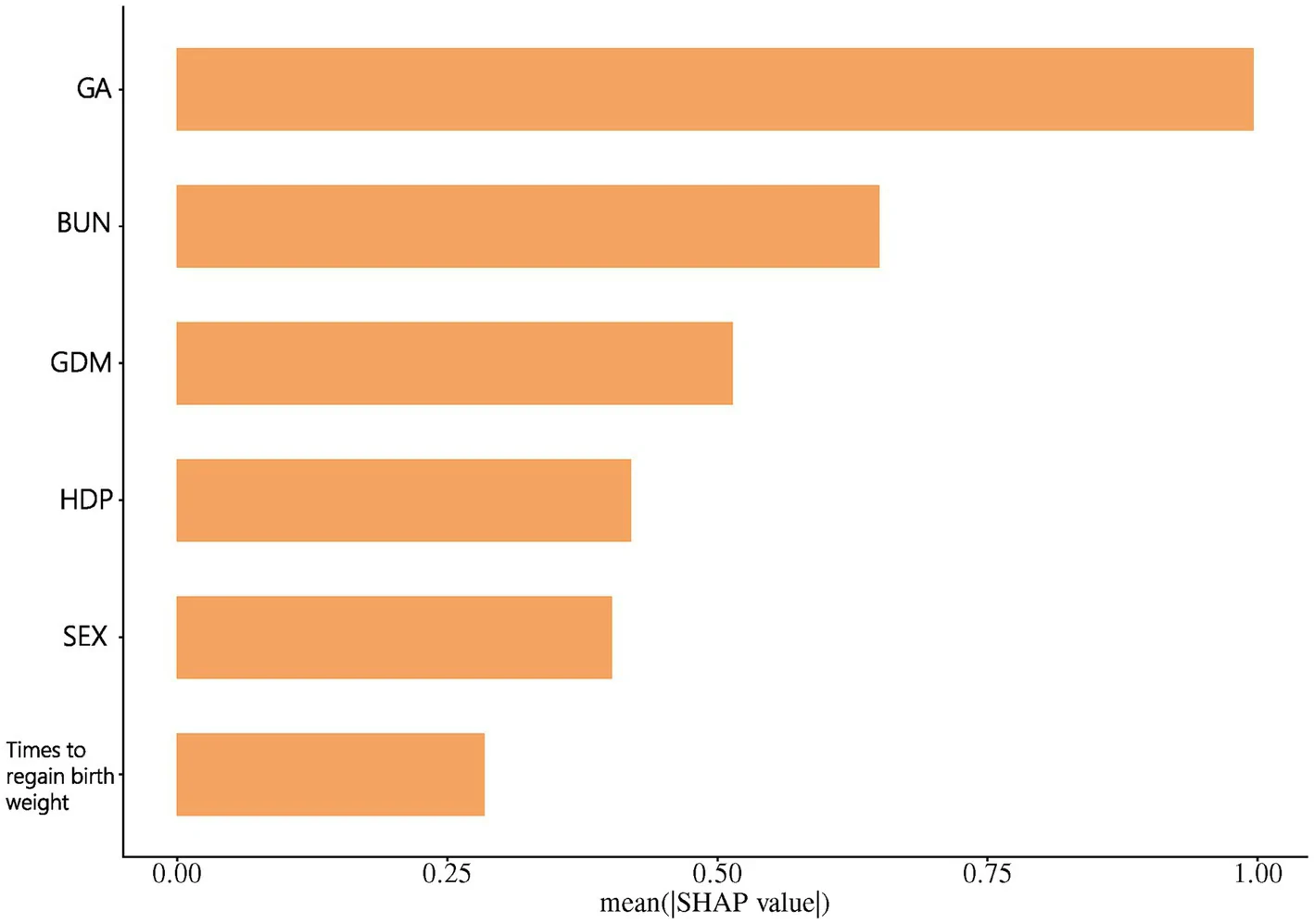
SHAP feature importance plot. BUN, blood urea nitrogen on day 14. GDM, gestational diabetes mellitus, HDP, hypertensive disorders of pregnancy, GA: Gestational age (W), Regain BW: Time to regain birth weight (d).

**Figure 7:**
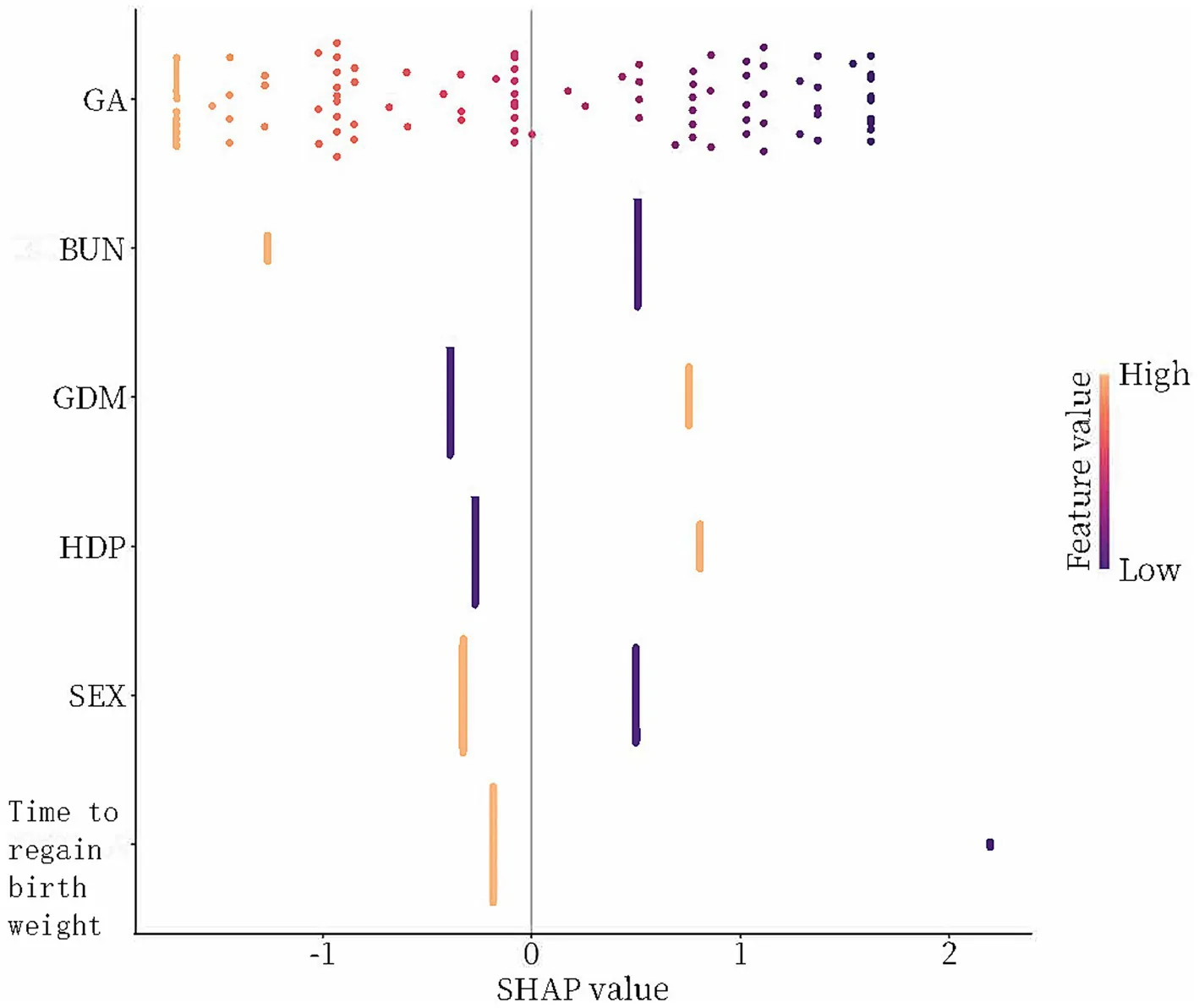
SHAP beeswarm plot of feature importance. BUN, blood urea nitrogen on day 14; GDM, gestational diabetes mellitus; HDP, hypertensive disorders of pregnancy; GA: Gestational age (W); Regain BW: Time to regain birth weight (d).

**Figure 8:**
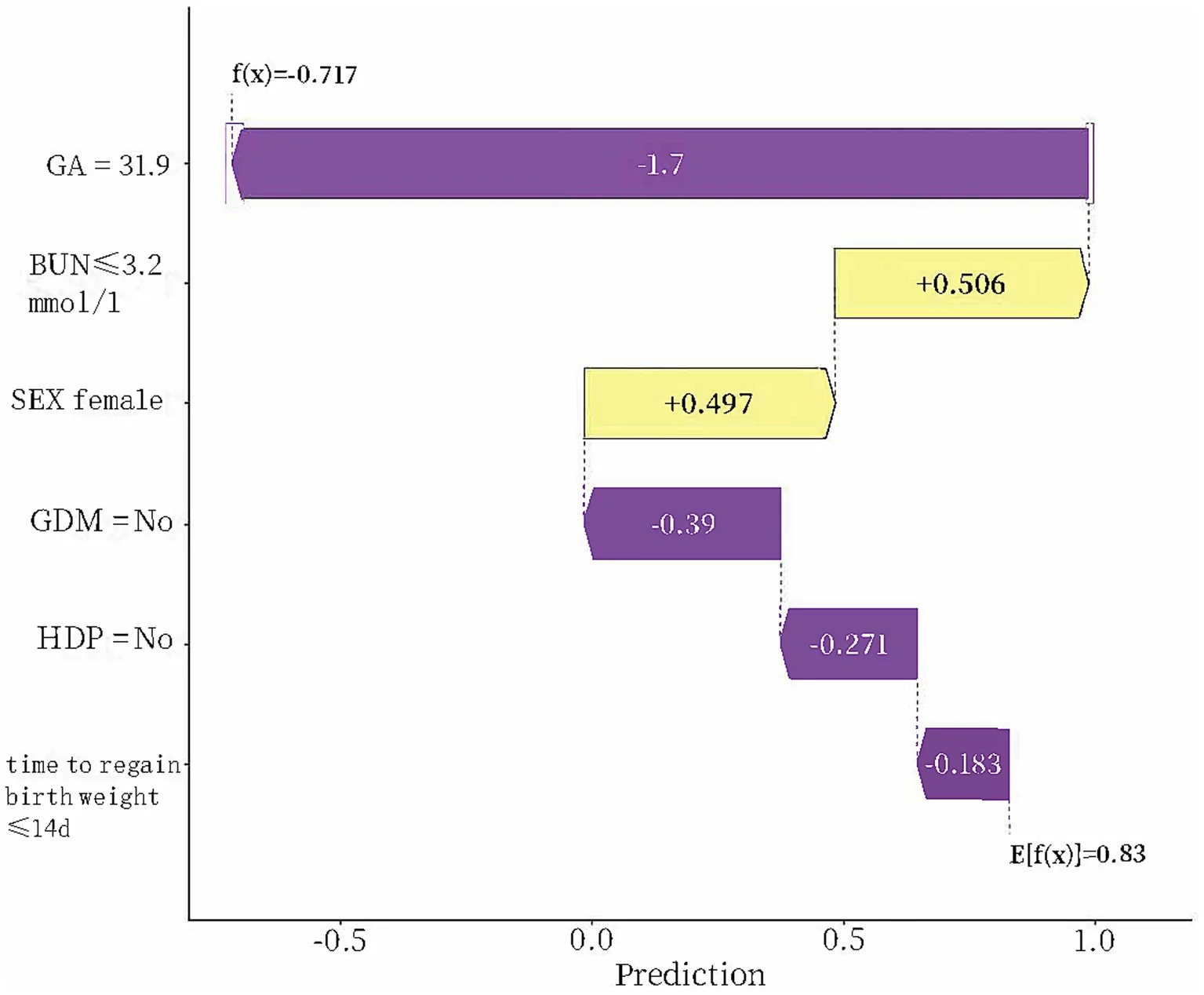
SHAP waterfall plot for logistic regression model illustrating the prediction explanation for a single sample (*n* = 100). Yellow bars indicate features that exert a positive influence on the predicted probability, whereas purple bars denote features with a negative influence. BUN, blood urea nitrogen on day 14; GDM, gestational diabetes mellitus; HDP, hypertensive disorders of pregnancy; GA: gestational age (W); Regain BW: time to regain birth weight (d).

To interpret the machine learning models, we selected the logistic model because it showed the best performance in the test cohort. The ranking of feature importance from highest to lowest was as follows: GA, BUN, GDM, HDP, sex, time to regain birth weight. SHAP values were further applied to provide transparent and interpretable estimates of individual feature contributions. The SHAP summary plot revealed the following ranking of diagnostic contribution: GA, BUN, GDM, HDP, sex, time to regain birth weight (shown in Figures 6, 7). To enhance comprehension of the model’s decision process at the individual level, we conducted a detailed interpretability analysis on one representative sample (case 100), as illustrated in Figure 8. By visualizing the SHAP values of the sample, we could discern the impact of each feature on the model’s predictions for these specific instances.

## 4 Discussion

By integrating logistic regression with machine learning techniques, we analyzed clinical data from the antenatal period through the first 14 postnatal days in very preterm infants to identify risk factors for longitudinal EUGR. This analysis enabled the development of an early prediction model, which we translated into a clinically applicable nomogram. Comparative validation against four other common machine learning algorithms confirmed that our logistic regression-based model achieved superior overall predictive performance. This practical tool holds the potential to help clinicians identify infants at high risk of longitudinal EUGR early on, enabling them to provide timely and personal nutritional interventions and ultimately improve their long-term outcomes.

The reported incidence of EUGR in preterm infants has ranged from 31.7 to 80% (34–36), which is largely attributable to the use of divergent diagnostic criteria and reference growth curves. Lara González-García et al. found that the incidence of EUGR based on the Fenton growth chart was higher than that based on the INTERGROWTH-21st chart, and that different diagnostic criteria also produced varying incidence rates. Regardless of the growth chart used, cross-sectionally defined EUGR incidence (73.8% with Fenton 2013; 59.3% with INTERGROWTH-21st) was higher than longitudinally defined incidence (44.3 and 29.3%, respectively) (37). Analysis of the U.S. Vermont Oxford Network database revealed divergent patterns: in non-SGA, very low birth weight infants, cross-sectional EUGR incidence (77.2%) was lower than longitudinal incidence (85.5%) when using the Fenton chart. Notably, this pattern reversed in SGA infants, with cross-sectional incidence reaching 99.7% compared to 43.7% for longitudinal definition (6).

Given that IUGR is a key determinant of SGA status, a high cross-sectional EUGR incidence in SGA preterm infants may predominantly reflect the persistence of prenatal growth restriction rather than an independent postnatal growth failure.

Nutritional management based on cross-sectional EUGR criteria may lead to excessive nutritional support and an increased risk of obesity (38). Consistent with previous evidence from a Chinese cohort of very preterm infants, our data demonstrated that the longitudinal EUGR definition (based on Z-score difference) was robust and independent of SGA status (39). In a sensitivity analysis excluding 29 SGA infants (Supplementary Table 2), the previously observed protective association of male sex was attenuated and no longer significant (OR 0.57, 95% CI 0.28–1.15, *p* = 0.116), yet the remaining five core predictors retained their statistical significance (all *p* < 0.05). These findings further support that our longitudinal definition of EUGR is unaffected by SGA status. Moreover, longitudinal criteria have been shown to be more sensitive than cross-sectional ones in identifying true postnatal growth failure and in predicting subsequent growth restriction at 2–2.5 years of age (12). Therefore, in the present study, the longitudinal definition of EUGR with reference to the Fenton growth curves was adopted, yielding an incidence of 65.4% in preterm infants born at 28–31^+6^ weeks’ gestation. This finding was consistent with reports from similar populations using comparable definitions, such as the 60% reported by the Chinese Neonatal Network (4) and the 58.4% reported in a Greek cohort (40). However, incidence was higher than the rate of 44.3–47.2% and 38–47% reported in larger European and Canadian cohorts, respectively (41, 42). This observed discrepancy underscores that while EUGR remains a prevalent global challenge, the relatively higher incidence identified in our study highlights an urgent need to enhance early risk prediction and optimize nutritional management from the earliest postnatal period to reduce the occurrence of EUGR.

Accurately predicting EUGR first requires identifying its high-risk factors. While traditional univariate or multivariate logistic regression clearly illustrates causal relationships and provides good interpretability for linear associations, it is often limited by sensitivity to sample size, data imbalance, and a failure to capture complex nonlinear relationships. Machine learning excels at handling large, multidimensional datasets, analyzing intricate associations less affected by multicollinearity, and identifying optimal clinical predictors. These capabilities have led to its successful application in diverse clinical settings for efficiently screening complex data (43, 44). To leverage the complementary strengths of traditional regression and machine learning for variable selection, we employed a dual approach. First, LASSO regression was applied to reduce model complexity and enhance generalizability by effectively shrinking coefficients and performing embedded feature selection. Enabling efficient screening of complex clinical data and uncovering intricate associations that were less affected by multicollinearity (45, 46). The final predictors included in our model were GA, sex, GDM, HDP, BUN, and time to regain birth weight. Spanning maternal health, neonatal characteristics, early weight dynamics, and metabolic-nutritional indicators, these variables facilitate a comprehensive, multi-domain assessment of EUGR risk.

In our study, as well as in prior literature (4, 47), low gestational age (GA) was consistently identified as a key risk factor for EUGR. This association was strongly corroborated by our machine learning models, in which GA emerged as the most influential predictor among all variables assessed via SHAP importance rankings. Cohort studies indicated that very preterm infants experience persistent growth restriction, often failing to catch up by term-equivalent age, unlike their moderate-to-late preterm counterparts who frequently achieve catch-up growth (48). The elevated risk is driven by a cascade of interrelated factors initiated by physiological immaturity: (1) Physiological and metabolic immaturity: Lower GA was directly associated with insufficient metabolic reserves and immature gastrointestinal function, leading to delays and interruptions in enteral feeding and, consequently, significant cumulative energy and protein deficits (49–52). (2) High burden of morbidities: this inherent vulnerability predisposes very preterm infants to a higher incidence of morbidities, such as BPD, late-onset sepsis, and NEC, which in turn exacerbate nutritional losses and metabolic demands (53–56). Interestingly, male was identified as a protective factor against the outcome in our cohort. While this direction is consistent with several previous reports (4, 16, 57). It contrasts with the widely recognized “male disadvantage” in neonatal morbidity and growth observed in most contemporary networks. We speculate that this discrepancy may partly reflect the borderline statistical significance and relatively wide confidence interval, which increase the risk of type I error or sampling fluctuation. In addition, the relatively narrow gestational age window (28–31^+6^ weeks) and the single-center design may have further amplified center-specific or period-specific effects. Therefore, this finding should be interpreted with caution and requires validation in larger, multi-center late-preterm populations.

Both GDM and HDP were retained as significant predictors in our final model, which is consistent with previous research (47). Notably, while preterm infants born before 32 weeks to mothers with GDM initially regained their birth weight slightly faster, they subsequently exhibited more pronounced early weight loss (58). This pattern suggested a potential impairment in postnatal metabolic adaptation among this vulnerable subgroup. The underlying pathophysiology linked GDM to offspring growth restriction primarily through placental dysfunction, which encompassed vascular lesions, metabolic disturbances, and immune imbalance. These detrimental effects were further exacerbated by the presence of concurrent maternal conditions, such as obesity, hyperlipidemia, or gestational hypertension (59, 60). Beyond early growth patterns, exposure to GDM was associated with an increased long-term risk of obesity, diabetes, and other metabolic disorders in offspring during adulthood (61). Collectively, these findings indicate that in very preterm infants, maternal GDM was associated with a biphasic weight trajectory: despite a slightly accelerated initial recovery of birth weight, it was followed by a more pronounced and clinically significant phase of weight loss, suggesting an underlying impairment in metabolic adaptation. Beyond metabolic dysregulation, another critical prenatal factor influencing preterm growth was placental vascular pathology. HDP, particularly preeclampsia and cases complicated by fetal growth restriction, significantly elevated the risk of EUGR in preterm infants. This association was biologically grounded in placental dysfunction, which encompassed inadequate perfusion, oxidative stress, and angiogenic imbalance. These pathological processes contributed to intrauterine growth restriction and low birth weight, thereby predisposing infants to EUGR (62–64). Preterm infants born to mothers with HDP faced higher incidences of morbidities of prematurity such as RDS, BPD, NEC and so on. These conditions further challenged postnatal growth by increasing metabolic demands for repair and homeostasis, impairing the establishment of enteral feeding, and necessitating clinical interventions that restricted nutrient delivery and utilization (e.g., mechanical ventilation, fasting), thereby directly contributing to EUGR (53, 65, 66).

BUN levels were recognized as a critical biomarker for assessing protein intake, providing robust support for early evaluation of nutritional status, according to breastfeeding guidelines, a BUN level below 3.2 mmol/L indicates inadequate protein intake, signaling the need for adjustments in nutritional management strategies (67–69). Miller et al. also suggested that persistently low BUN values indicate a risk of undernutrition (70). Therefore, we introduced BUN as an indicator to assess the risk of EUGR. Given the relatively small sample size of this study, which may limit its generalizability, we used the guideline-recommended threshold as the cut-off value for this predictor and combined it with other indicators in a joint prediction model to improve predictive performance. In our model, SHAP analysis identified BUN as the second most important predictor, with a clear inverse relationship to EUGR risk, highlighting its significant predictive value. This observation aligned with earlier studies linking EUGR to a significantly negative nitrogen balance in early life (71).

The present study also found that prolonged time to regain birth weight was an independent risk factor for EUGR in preterm infants, serving as a direct marker of early nutritional insufficiency (35, 36). These findings aligned with previous report linking delayed weight recovery to higher EUGR risk at discharge (72). This delay, frequently linked to interruptions in enteral feeding or sepsis, served as the strongest predictor of subsequent growth velocity, with each additional day required to regain birth weight corresponding to a significant reduction in growth rate, particularly in infants with slower overall growth (73). Therefore, monitoring the time to regain birth weight alongside BUN was prioritized as early, critical indicators for EUGR risk assessment and nutritional management.

By integrating multidimensional predictors via machine learning or statistical algorithms, prediction models can establish robust decision rules to enhance early disease diagnosis. A multicenter Chinese study of 2,514 preterm infants (<32 weeks’ gestation) developed a logistic regression model for cross-sectionally defined EUGR, incorporating predictors including birth weight, multiple birth, SGA status, maternal complications (HDP, GDM), and nutritional/clinical factors (cumulative fasting duration, growth velocity, postnatal corticosteroids). The model demonstrated robust discriminative ability, with AUCs of 0.831 (training) and 0.846 (validation), and was translated into a clinical nomogram (47). Xie et al. analyzed data from 387 preterm infants with a gestational age of ≤34 weeks with cross-sectional definition of EUGR, identified six key predictors using Lasso regression (gestational age, birth weight, premature rupture of membranes, patent ductus arteriosus, cholestasis, and neonatal sepsis), and subsequently developed random forest, support vector machine, and logistic regression models. Among these, the random forest model showed the best performance with an AUC of 0.62 and an accuracy of 72.7% (74). A Greek prediction model, developed using XGBoost on a cohort of 650 preterm infants (<33 weeks’ gestation), employed a longitudinal EUGR definition and constructed stratified models for different gestational age groups, achieving good discriminative performance (40). However, the model did not incorporate maternal antenatal complications and underwent neither further calibration nor external validation. Huang Xiaofang et al. developed a longitudinal EUGR nomogram for extremely preterm infants (<28 weeks’ gestation) based on seven variables, demonstrating good discrimination (75). However, a pivotal limitation is that the model’s prediction time point falls beyond the critical early postnatal window, thereby precluding its use for guiding proactive nutritional optimization when interventions could be most impactful. EUGR represents a progressive growth deviation rather than an acute event, making its prevention dependent on early risk identification to guide timely, targeted nutritional support. To address this, our study developed a model specifically for early prediction. We adopted the longitudinal definition of EUGR and restricted all predictors to the first 14 postnatal days, a critical window that captures the pivotal transition from parenteral to enteral nutrition. Through a rigorous, multi-stage variable selection process integrating univariate logistic regression and LASSO, the model incorporates a comprehensive set of neonatal and maternal characteristics. A key methodological advance is the inclusion of BUN, a directly measured nutritional biomarker absent from all prior EUGR prediction models. By quantifying protein metabolism during this metabolic shift, BUN enhances the biological plausibility of the model. Furthermore, we performed specific risk stratification within the 28–31^+6^ weeks gestational age subgroup, providing the first tailored risk assessment for these moderately preterm infants during this vulnerable period. The resultant tool enables evidence-based, early identification of EUGR risk, thereby facilitating timely and personalized nutritional management to improve long-term outcomes.

This study conducted a comprehensive comparison between the performance of the logistic regression model and several commonly used machine-learning models. Four machine-learning algorithms showed good predictive performance in the testing phase, among which CatBoost achieved the highest predictive accuracy. The logistic regression model demonstrated superior overall performance in the validation cohort. While its AUC was slightly lower than that of CatBoost, it achieved higher diagnostic accuracy, positive predictive value, and F1 score. This balanced performance profile, when translated into an intuitive nomogram, afforded greater clinical practicality and interpretability. Although machine-learning models may enhance predictive accuracy, their “black-box” nature limited transparency in decision pathways. In contrast, the linear structure of logistic regression allowed direct interpretation of feature weights, offered clear quantification of variable effects such as odds ratios, and facilitated clinical understanding, making it a valuable tool for diagnostic and decision-making purposes (76, 77). However, our decision curve analysis demonstrated net benefit at threshold probabilities of 25–95% in the training set and 35–95% in the test set. Although these thresholds exceed those typically applied in clinical nutrition, this discrepancy aligns with the low-risk, low-cost nature of nutritional interventions. Clinicians often initiate treatment at 10–20% thresholds; however, the model showed limited net benefit in this range. Given the minimal harm associated with overtreatment, even marginal net benefit may yield population-level advantages by broadening risk identification. Future recalibration incorporating nutritional care costs is required to optimize utility at these lower thresholds.

Several limitations warrant consideration. First, the testing cohort was relatively small (*n* = 78), yielding a broad 95% CI for the AUC (0.713–0.914). The substantial overlap between this interval and that of the machine learning model precludes definitive conclusions regarding absolute superiority. Second, our internal split-sample validation constitutes the lowest tier of evidence. The absence of external validation raises concerns about generalizability, particularly given the well-documented performance degradation of prediction models in external cohorts. Third, the BUN cutoff (3.2 mmoL/L) was adopted from interventional literature rather than derived empirically from this cohort. While this approach may enhance clinical transportability, it might not represent the optimal threshold for this specific population. Fourth, time to regain birth weight was dichotomized at 14 days according to clinical convention. The number of patients with regain time >14 days in the non-EUGR group was small, and this limited sample size may affect the precision of the coefficient estimate. Although this variable received relatively high points in the nomogram, this is partly attributable to mathematical scaling effects; its actual weighting should be interpreted in conjunction with the overall model and requires further clarification through external validation. Finally, our EUGR definition relies exclusively on weight gain. Head growth is a stronger predictor of long-term neurodevelopmental outcomes, our weight-based metric may fail to capture the nuanced risk profiles relevant to neurological prognosis. To address these limitations and robustly evaluate the model’s clinical utility, future prospective, multicentre studies with larger cohorts were warranted.

## 5 Conclusion

We developed and validated a logistic regression model to predict longitudinal EUGR in very preterm infants. The model demonstrated strong predictive accuracy, good calibration, and positive net clinical benefit upon internal validation. Notably, it outperformed four comparative machine learning algorithms in predictive performance. Furthermore, a visualized interpretation of the model quantified the individual risk contribution of each predictor, translating it into a simple, intuitive, and practical tool for clinicians to facilitate individualized early risk assessment and intervention in this vulnerable population.

## Data Availability

The raw data supporting the conclusions of this article will be made available by the authors, without undue reservation.
