# Supplementary material for: Predicting extrauterine growth restriction at the parenteral-enteral nutrition transition: a model for risk identification in very preterm infants
Source: Front Nutr. 2026 Sep 3;13:1788647. doi: 10.3389/fnut.2026.1788647 (PMC13581580; doi:10.3389/fnut.2026.1788647)
Supplement: Supplementary file 1 [file Table_1.DOCX]

Supplementary Material

# 1 Supplementary Tables

# Supplementary Table 1 Inter-model comparison using DeLong’s Test

| Model_A | Model_B | AUC_A | AUC_B | Z_Statistic | *P* |
| --- | --- | --- | --- | --- | --- |
| KNN | CatBoost | 0.740 | 0.820 | -2.0866 | 0.0369 |
| LM | KNN | 0.814 | 0.740 | 2.045 | 0.0409 |
| SVM | CatBoost | 0.774 | 0.820 | -1.7194 | 0.0855 |
| LM | SVM | 0.814 | 0.774 | 1.6278 | 0.1036 |
| LightGBM | CatBoost | 0.787 | 0.820 | -1.3663 | 0.1719 |
| KNN | LightGBM | 0.740 | 0.787 | -1.204 | 0.2286 |
| KNN | SVM | 0.740 | 0.774 | -1.1445 | 0.2524 |
| LM | LightGBM | 0.814 | 0.787 | 0.7952 | 0.4265 |
| SVM | LightGBM | 0.774 | 0.787 | -0.4429 | 0.6578 |
| LM | CatBoost | 0.814 | 0.820 | -0.2837 | 0.7767 |

Abbreviations: LM, logistic regression model; SVM, support vector machine; KNN,K-nearest neighbors; LightGBM, light gradient boosting machine

# Supplementary table 2 Sensitivity analysis of EUGR: comparison between overall cohort and cohort excluding SGA infants.

|  | EUGR (excluded SGA) | | EUGR | |
| --- | --- | --- | --- | --- |
| characteristics | OR(CI) | *P* | OR(CI) | *P* |
| Sex-male | 0.57(0.283-1.148) | 0.116 | 0.44 (0.20 ~ 0.97) | 0.043 |
| GA | 0.442(0.328-0.597) | <.001 | 0.09 (0.01 ~ 0.81) | <.001 |
| Time to Regain Birth Weight | 0.132(0.027-0.650) | 0.013 | 0.43 (0.30 ~ 0.60) | 0.032 |
| GDM | 4.198(1.982-8.893) | <.001 | 3.14 (1.35 ~ 7.28) | <.001 |
| HDP | 2.857(1.199-6.809) | 0.018 | 2.93 (1.13 ~ 7.59) | 0.027 |
| BUN-14 | 0.159(0.073-0.346) | <.001 | 0.17 (0.07 ~ 0.42) | <.001 |

Abbreviations: BUN-14, blood urea nitrogen on day 14; GDM, gestational diabetes mellitus; HDP, hypertensive disorders of pregnancy; GA, gestational age.

# Supplementary table 3 Multivariable analysis of BUN as an independent predictor

| Characteristic（n=260） | β | SE | OR(CI) | Z | *P* | Sensitivity | Specificity |
| --- | --- | --- | --- | --- | --- | --- | --- |
| BUN-14 | 0.898 | 0.281 | 0.407(0.235-0.707) | -3.198 | ＜0.001 | 0.7391 | 0.4328 |

Abbreviations: BUN-14, blood urea nitrogen on day 14

# 2 Supplementary Figures

**Supplementary Figure 1.** Confusion matrices for five models: (A) KNN, (B) CatBoost, (C) SVM, (D) Logistic Regression, and (E) LightGBM.

**Supplementary Figure 2.** Unadjusted ROC Curves for BUN Using a Cutoff of 3.2 mmol/L
